# Psychoacoustic characteristics of tinnitus in temporomandibular disorders: a systematic review

**DOI:** 10.1590/2317-1782/e20250343en

**Published:** 2026-07-27

**Authors:** Taís de Azevedo Picinini, Ingrid Eismann Moraes, Karen Dantur Batista Chaves, Alexandre Hundertmarck Lessa, Adriana Laybauer Silveira Unchalo, Adriane Ribeiro Teixeira

**Affiliations:** 1 Programa de Pós-graduação em Saúde da Comunicação Humana, Universidade Tuiuti do Paraná – UTP - Curitiba, PR, Brasil.; 2 Curso de Fonoaudiologia, Universidade Federal do Rio Grande do Sul – UFRGS - Porto Alegre (RS), Brasil.; 3 Departamento de Odontologia Conservadora, Faculdade de Odontologia, Universidade Federal do Rio Grande do Sul – UFRGS - Porto Alegre (RS), Brasil.; 4 Departamento de Saúde e Comunicação Humana, Instituto de Psicologia, Serviço Social, Saúde e Comunicação Humana, Universidade Federal do Rio Grande do Sul – UFRGS - Porto Alegre (RS), Brasil.; 5 Serviço de Fonoaudiologia, Hospital de Clínicas de Porto Alegre – HCPA - Porto Alegre (RS), Brasil.

**Keywords:** Audiology, Hearing, Psychoacoustics, Temporomandibular Joint Dysfunction Syndrome, Tinnitus

## Abstract

**Purpose:**

To describe the psychoacoustic characteristics of tinnitus in patients with temporomandibular disorders (TMD).

**Research strategies:**

A systematic review was conducted following PRISMA guidelines, with the protocol registered in PROSPERO (CRD42025649038). Searches were performed in Embase, LILACS, PubMed/MEDLINE, Scopus, Web of Science, and Google Scholar, using validated MeSH and DeCS terms.

**Selection criteria:**

Descriptive, observational, cross-sectional, case-control, or longitudinal studies evaluating patients with TMD and tinnitus, reporting psychoacoustic parameters such as pitch and loudness, were included. Studies involving animals, single-case reports, case series, reviews, letters, editorials, or articles without original data were excluded.

**Data analysis:**

Two reviewers independently screened titles, abstracts, and full texts, with a third reviewer resolving disagreements. Risk of bias was assessed using the JBI Critical Appraisal Tools. Data were extracted, organized, and qualitatively synthesized.

**Results:**

A total of 271 articles were identified; after removal of duplicates and eligibility assessment, five studies were included in the synthesis. Findings indicate that tinnitus in TMD patients is predominantly unilateral, continuous, with high pitch and moderate loudness. Some patients can modulate tinnitus through mandibular or cervical movements, suggesting a somatosensory contribution. The impact on quality of life ranges from mild to moderate, even in individuals with normal hearing.

**Conclusion:**

Tinnitus associated with TMD shows relatively consistent psychoacoustic characteristics. However, further primary studies with larger sample sizes and robust designs are needed to confirm specific patterns and improve multidisciplinary therapeutic approaches.

## INTRODUCTION

Tinnitus is an auditory sensation that is not associated with an external sound source and generally causes substantial discomfort, often resulting in impaired quality of life. It is a symptom that may arise from several conditions and pathologies, among which temporomandibular disorder (TMD) has been identified as a relevant associated factor^(1)^. Tinnitus is commonly perceived and described as a ringing or whistling sound in the ears and/or head. Although it is frequently referred to as “ringing in the ears,” this condition may manifest in different forms, including ringing itself, as well as sounds such as swooshing and clicking^(2,3)^.

Regarding symptom duration, tinnitus may be classified as acute when it persists for a short period, such as after exposure to high-intensity sound during a concert. It may also be considered a chronic health condition when perceived continuously and persistently by the individual, or it may occur intermittently. Furthermore, while some patients report tinnitus as an extremely disabling condition, others describe the sensation as nearly imperceptible^(2-4)^.

Tinnitus may be triggered by several conditions, including noise-induced hearing loss, presbycusis, the use of ototoxic medications, metabolic disorders, and musculoskeletal alterations, including temporomandibular disorders (TMD), which have been increasingly recognized as factors associated with both the generation and modulation of this symptom.

Temporomandibular disorder is a condition encompassing a heterogeneous group of musculoskeletal and neuromuscular disorders involving the temporomandibular joint (TMJ), the masticatory muscles, and adjacent structures. Its signs and symptoms may include joint sounds, alterations in mandibular function, and pain^(5)^. According to the American Academy of Orofacial Pain, TMD is defined as a group of disorders involving the masticatory muscles, the temporomandibular joint (TMJ), and associated structures^(6)^.

Although the association between TMD and tinnitus has been widely recognized, the specific psychoacoustic characterization of tinnitus in these patients, particularly regarding pitch and loudness, as well as its correlations with audiological findings obtained through examinations such as pure-tone audiometry and immittance testing, remains inconsistent across studies. To date, no systematic review has integrated these findings comprehensively. This gap justifies the present review, which aims to synthesize the available evidence and describe the psychoacoustic and audiological characteristics of tinnitus in individuals with TMD, seeking to identify clinical patterns that may guide future investigations and support the development of more targeted therapeutic strategies.

## METHODS

### Search strategies and eligibility criteria

The research question was defined as follows: “What are the psychoacoustic characteristics of tinnitus in patients with temporomandibular disorder (TMD)?” The PECOS strategy (Population, Exposure, Comparison, Outcome, and Study design) adopted for this review was structured as follows:

P (Population): patients with TMD;

E (Exposure): presence of tinnitus;

C (Comparison): any other group of individuals with tinnitus and without TMD;

O (Outcome): tinnitus characteristics;

S (Study design): descriptive, observational, cross-sectional, case-control, or longitudinal studies

The review was conducted in accordance with the *Preferred Reporting Items for Systematic Reviews and Meta-Analyses* (PRISMA) guidelines^(7)^ and followed the stages recommended in the *Cochrane Handbook for Systematic Reviews* ^(8)^, including formulation of the research question, identification and selection of studies, critical appraisal of methodological quality, data extraction, analysis and presentation of findings, interpretation of results, and, finally, review refinement and updating. The protocol for the present review was registered in the PROSPERO database (CRD42025649038).

The databases searched were Embase, Latin American and Caribbean Health Sciences Literature (LILACS), PubMed/MEDLINE, Scopus, Web of Science, and Google Scholar for gray literature.

Database searches were conducted in September 2024, and the final update was performed on October 14, 2024, in the following databases: Embase, Latin American and Caribbean Health Sciences Literature (LILACS), PubMed/MEDLINE, Scopus, Web of Science, and Google Scholar. All search strategies were developed and reviewed by two experienced researchers. The descriptors used were verified in the controlled vocabularies Medical Subject Headings (MeSH) and Health Sciences Descriptors (DeCS), accessed through the CAPES Journal Portal^(9)^. Based on these descriptors, specific search strategies were developed for each database by combining the selected terms, as detailed in Appendix A. No filters regarding study design, language, or publication date were applied. No contact was made with authors to obtain additional data.

The Google Scholar search was performed to identify relevant gray literature. No language restrictions were applied. The page settings were adjusted to display 20 results per page, and the first 100 results (five pages), ranked by relevance, were screened. The command *‘filetype:pdf’* was used to restrict the search to *Portable Document Format* (PDF) files with full-text availability. Screening of the results was conducted through title and abstract reading, and only studies meeting the predefined eligibility criteria were included.

Although the synthesis of findings was descriptive, the present study retained a systematic review design because it fully complied with all PRISMA-recommended stages, including a structured search strategy, clearly defined eligibility criteria, and critical appraisal of the methodological quality of the included studies.

## SELECTION CRITERIA

The studies were independently selected and reviewed by two evaluators according to the predefined inclusion and exclusion criteria. The Kappa agreement coefficient between reviewers was higher than 0.7, indicating a high level of inter-reviewer agreement.

### Inclusion criteria

Studies published in Portuguese, English, or Spanish were included, with no restriction regarding publication date. Eligible studies involved patients of any age group who underwent some type of auditory assessment and presented tinnitus associated with TMD. The studies were required to report psychoacoustic tinnitus data, such as pitch, loudness, or other descriptive characteristics. Observational, descriptive, cross-sectional, and longitudinal studies addressing the outcome of interest in this review were considered eligible.

### Exclusion criteria

Articles that did not report psychoacoustic characteristics of tinnitus in TMD were excluded, as were case reports, case series, literature reviews, letters to the editor, editorials, opinion articles without original data, and studies using animal models.

## STUDY SELECTION AND DATA ANALYSIS

Data were extracted according to the search strategies defined for each database, exported in RIS format, and organized using the Zotero reference management software. Subsequently, the records were imported into the Rayyan screening platform, where Phase 1 was initiated. During this stage, two reviewers independently screened the titles and abstracts of all retrieved studies, excluding those that did not meet the established criteria. In cases of uncertainty regarding eligibility, the full text of the article was assessed. In Phase 2, the same reviewers independently conducted full-text assessments of potentially eligible studies. Whenever disagreements occurred between reviewers, a third reviewer acted as an arbitrator.

### Data extraction and synthesis

Data extraction from the studies selected for synthesis was independently performed by the two reviewers. For each included study, the following information was collected: author, year of publication, country, study design, sample size and characteristics, instruments and methods used for auditory assessment and for measuring the psychoacoustic characteristics of tinnitus (pitch, loudness, type of stimulus, laterality, among others), main findings, and conclusions.

Discrepancies in data extraction were discussed until consensus was reached, with mediation by a third reviewer when necessary. The synthesis of findings was conducted descriptively and qualitatively, considering the variables most frequently reported across the studies. Results were organized into extraction tables and analyzed according to the trends observed in psychoacoustic measures and their possible relationships with audiological findings.

## RESULTS

### Selection of evidence sources

**Figure 1:**
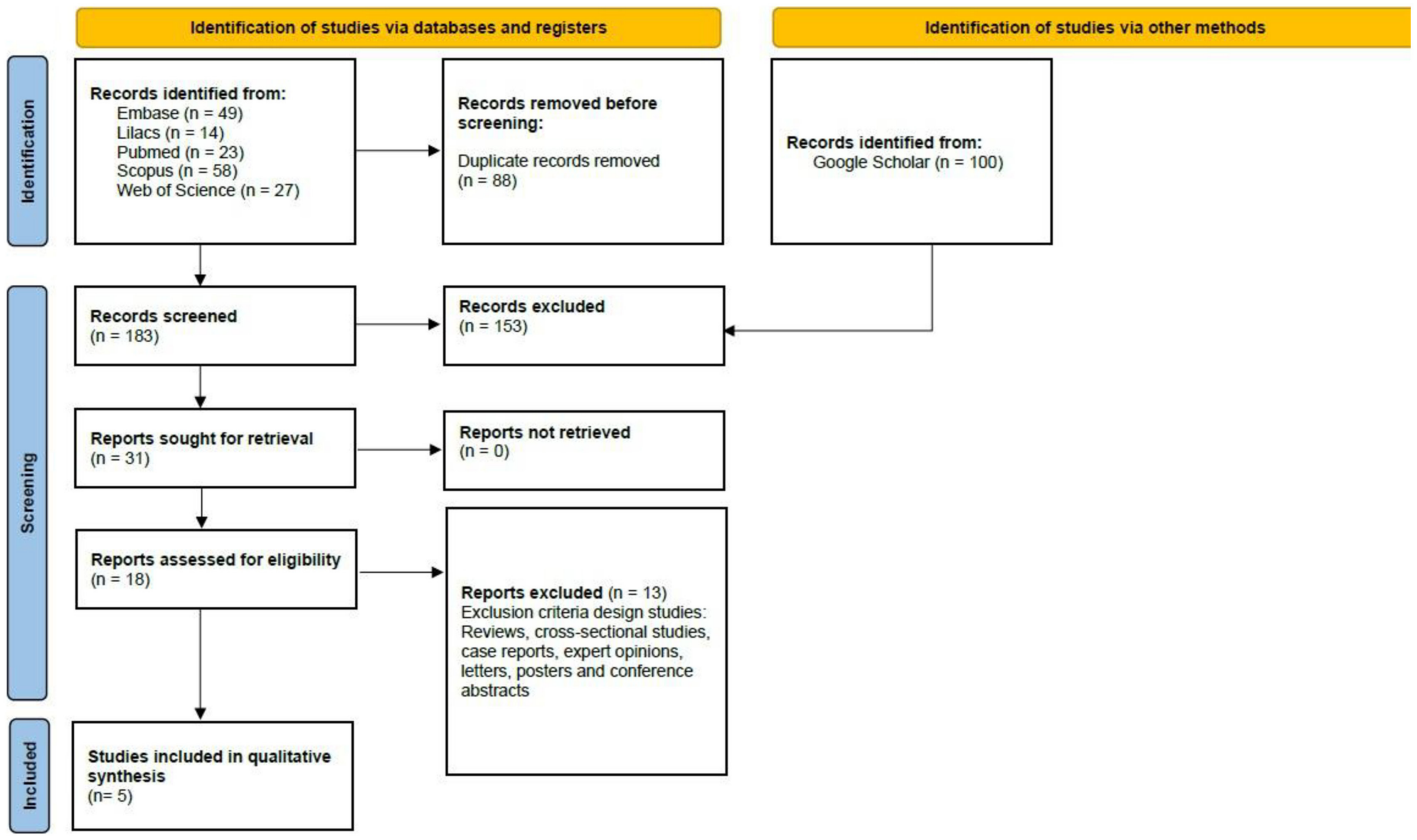
PRISMA flow diagram of the selection process for articles included in the systematic review

Initially, 271 articles were identified through the electronic database searches. Of these, 137 were duplicates, among which 49 were resolved and 88 were excluded. After duplicate removal, the titles and abstracts of 183 articles were screened according to the predefined inclusion and exclusion criteria, resulting in the exclusion of 153 articles. Ten studies remained in conflict and were referred to a third reviewer for arbitration. Subsequently, 18 articles were selected for full-text reading, of which five studies were ultimately included for analysis and synthesis (Figure 1). Appendix B presents the reasons for exclusion of each study not included in the present review.

*From:* Page et al.^(7)^. For more information, visit the PRISMA website^(29)^

### Results of individual sources of evidence

The included studies were published between 2011 and 2019 and comprised Brazilian studies^(10-12)^, as well as investigations conducted in South Africa and Germany^(13,14)^. Table 1 summarizes the data extracted from the included studies, including author, year of publication, country of origin, study design, sample characteristics, age and sex of participants, objectives, assessment procedures, tinnitus characteristics evaluated, main findings, and conclusions.

**Table 1:** Data extraction from the included studies (N = 5)

**Author and Year (Country/Study Design)**	**Sample, Age, and Sex**	**Objective**	**Assessment**	**Evaluated Tinnitus Characteristics**	**Results**	**Conclusion**
			**Procedures**			
Cassol et al.^(10)^, 2019 (Brazil) / Descriptive observational cross-sectional study	53 patients: 7 males and 46 females; ages ranging from 22 to 69 years, with a mean age of 41 years.	To investigate the auditory health of individuals with tinnitus associated with TMD during the pre-dental treatment stage.	Conventional pure-tone audiometry (PTA)	Tinnitus laterality	Tinnitus showed a strong association with the side affected by TMD, occurring on the same side in 52 out of the 53 participants analyzed (98%). When bilateral TMD was observed, tinnitus was also bilateral and more intense on the side where TMD was more severe.	All patients with TMD, regardless of the presence of tinnitus, should be referred for audiological monitoring.
			Speech audiometry	Aural fullness and otalgia	Unilateral tinnitus was reported by the majority of participants and was associated with the presence of aural fullness (77%) and absence of otalgia (53%).	
			Acoustic immittance measures		Most participants presented bilateral hearing thresholds within normal limits (RE = 43%; LE = 50%), followed by moderate hearing loss (RE = 30%; LE = 25%), as well as poorer performance in high-frequency audiometry (HFA) compared with conventional pure-tone audiometry (PTA).	
			High-frequency audiometry (HFA)		Fifty percent of the participants showed absence of transient evoked otoacoustic emissions (TEOAE), despite normal conventional audiometry findings.	
			Otoacoustic emissions (OAE)			
Kanji and Khoza-Shangase^(13)^, 2013 (South Africa) / Exploratory, comparative, non-experimental pilot study	20 participants:	To identify the clinical signs and symptoms of tinnitus in patients with TMD.	Questionnaire (adapted from the tinnitus questionnaire of the Carolina Ear and Hearing Clinic [2005]) related to tinnitus perception and the impact of tinnitus on the individual’s lifestyle.	*Pitch, loudness,* tinnitus onset, duration, and location	In the group without TMD (Group B), the majority of participants (8 out of 10) reported tinnitus with constant frequency and duration, whereas in the group with TMD (Group A), only 3 out of 10 reported this pattern. All participants in Group A described tinnitus as a single sound, whereas three participants in Group B reported multiple sounds. The sounds most frequently reported in Group A were “ringing” and “whistling,” whereas in Group B the predominant descriptions were “ringing” and “buzzing.”	The characteristics of tinnitus in patients with TMD differed from those observed in patients without TMD.
	Group A with TMD: 10 participants (1 male and 9 females)		Conventional pure-tone audiometry (PTA)		Regarding loudness, no statistically significant difference was observed between the groups; however, the group with TMD presented lower mean loudness (25.4 dB) compared with Group B (33.5 dB). Concerning pitch, eight participants in Group A matched their tinnitus to 6 kHz (6 with pure tone and 2 with narrowband noise). In the group without TMD, half of the participants matched tinnitus to 8 kHz, three to 1 kHz, one to 3 kHz, and one to 250 Hz narrowband noise.	Tinnitus may occur in patients with TMD and may present as high-frequency tinnitus, highlighting the importance of careful evaluation in patients with tinnitus, as this may have implications for both diagnosis and clinical management.
	Group B without TMD: 10 participants (5 males and 5 females)		Speech audiometry			
			Acoustic immittance measures			
			Psychoacoustic tinnitus measurements			
Lacerda et al.^(12)^, 2016 (Brazil) / Descriptive observational cross-sectional study	19 individuals with TMD, with a predominance of females in the sample (89.47%).	To evaluate the impact of tinnitus on the quality of life of patients with TMD.	Pure-tone audiometry, acuphenometry, and the Brazilian version of the THI questionnaire.	Time since symptom onset, sound description, location, type, duration, and frequency	A total of 63.12% of participants had experienced tinnitus for less than five years, and 15.79% described tinnitus sounds resembling whistling, rain, and/or crickets.	
	The mean age was 53.5 years (range: 26 to 72 years).				Regarding laterality, there was a predominance of right-ear tinnitus (42.11%), and 52.63% of participants reported continuous tinnitus.	A significant correlation was observed between THI scores, tinnitus duration, and frequency of occurrence.
					Audiometric evaluation demonstrated mean hearing thresholds above 25 dB HL at frequencies of 3000, 4000, 6000, and 8000 Hz bilaterally.	The tinnitus reported by patients with TMD caused a moderate impact on quality of life and could be perceived even in the presence of background noise, although daily activities could still be performed.
					Acuphenometry revealed a mean tinnitus loudness of 21 dB SL in the right ear and 17.85 dB SL in the left ear, whereas the mean tinnitus pitch was 3775 Hz in the right ear and 3750 Hz in the left ear.	
					The total THI score was 37.8 points.	
		
Vielsmeier et al.^(14)^, 2011 (Germany) / Case-control study	30 patients with a confirmed diagnosis of TMD and tinnitus compared with a group of 61 patients with tinnitus but without subjective complaints of TMD.	To investigate differences in clinical and demographic characteristics between the groups.	Otorhinolaryngological evaluation, including otoscopy and pure-tone audiometry.	Age at onset, type of onset (gradual or abrupt), type of tinnitus (pulsatile or non-pulsatile), loudness, tinnitus awareness/perception, masking, somatosensory modulation, hyperacusis, and tinnitus-related handicap assessed through the THI	Patients with tinnitus and TMD presented better auditory function (P < 0.0005), lower age (P = 0.001), and earlier tinnitus onset age (P = 0.002), in addition to being more frequently female (P = 0.003). These patients also reported lower subjective tinnitus loudness (P = 0.01) and greater ability to modulate tinnitus through jaw or neck movements (P = 0.001).	Classical risk factors for tinnitus (age, male sex, and hearing loss) appear to be less relevant in patients with tinnitus and TMD, suggesting a causal role of TMJ pathology in the generation and maintenance of tinnitus. Based on this finding, treatment of TMD may represent a causally oriented therapeutic strategy for tinnitus.
	Mean age of the total sample: 49.1 years.		Tinnitus Sample Case History Questionnaire and the German version of the Tinnitus Handicap Inventory (THI).		Furthermore, perceived tinnitus loudness was higher among patients without TMD (P = 0.01; mean difference: 13.1; 95% CI: 3.1–23.1).	
	56 males (61.5%) and 35 females (38.5%).				The remaining evaluated variables did not demonstrate significant differences between groups.	
		
Morais and Gil^(11)^, 2012 (Brazil) / Descriptive observational cross-sectional study	To characterize tinnitus in individuals with normal hearing and investigate its relationship with TMD.	20 adults of both sexes (70% female) with complaints of tinnitus and hearing thresholds within normal limits.	Audiological and otological history, acuphenometry, and application of a checklist of TMD signs and symptoms, developed by the researcher based on data from the literature, consultation with professionals in the field, and the Tinnitus Handicap Inventory (THI) questionnaire.	Investigation of tinnitus laterality, type, and duration, as well as identification of pitch and loudness.	Most participants reported high-pitched (75%), continuous (90%), and bilateral (60%) tinnitus. Acuphenometry revealed a mean pitch of 8.6 kHz and a mean loudness of 14.1 dB SL. Tinnitus-related distress was classified as mild, with a mean THI score of 25 points.	The most frequent tinnitus profile was characterized as high-pitched, continuous, and bilateral; 90% of participants presented at least one sign or symptom of TMD, and no correlation was observed between tinnitus and acuphenometry, THI scores, or the TMD checklist.
					Regarding TMD, 90% of participants presented at least one sign or symptom, with asymmetric mouth-opening movement being the most frequent finding (70%).	
		The age of the sample ranged from 20 to 55 years, with a mean age of 32.1 years.			A statistically significant correlation was observed between tinnitus pitch and loudness (the higher the pitch, the lower the loudness – P = 0.002), as well as between pitch and THI score (P = 0.002; the higher the pitch, the higher the THI score). No significant correlations were observed between findings from the TMD checklist and the psychoacoustic tinnitus parameters or THI scores, suggesting that, despite the high prevalence of TMD signs and symptoms in the sample, no direct relationship was identified between tinnitus characteristics and TMD.	
	

Caption: dB HL = decibels hearing level; dB SL = decibels sensation level; TMD = Temporomandibular Disorder; RE = Right Ear; LE = Left Ear; THI = Tinnitus Handicap Inventory

The studies selected for synthesis consistently confirmed that tinnitus is a common complaint among patients with TMD, even in the absence of hearing loss. One of the included studies^(10)^ reported that most patients with TMD presented unilateral tinnitus, generally associated with a sensation of aural fullness, although without otalgia. Despite normal hearing thresholds, alterations were identified in complementary audiological examinations, such as otoacoustic emissions. These findings reinforce the need for audiological follow-up even in patients without hearing loss.

Another study^(13)^ identified important differences in tinnitus characteristics between patients with and without TMD. Individuals with TMD tended to perceive tinnitus as a single sound rather than multiple simultaneous sounds, with lower loudness and higher pitch, generally around 6 kilohertz (kHz). Furthermore, these patients more frequently reported somatosensory modulation of tinnitus, meaning that they were able to alter tinnitus perception through mandibular or cervical movements, suggesting the involvement of the somatosensory system in the generation of the symptom. Similarly, other authors^(12)^ observed that most participants reported continuous tinnitus, predominantly localized in the right ear, with a mean pitch of approximately 3,750 Hz and mean loudness ranging between 17 and 21 decibels sensation level (dB SL). Scores obtained through the *Tinnitus Handicap Inventory* (THI) indicated a moderate impact on quality of life, with greater discomfort observed in individuals presenting tinnitus of longer duration and higher frequency of occurrence. A positive correlation was also observed between tinnitus duration and THI scores.

Researchers^(11)^ who investigated individuals with tinnitus and normal hearing reported that 90% of participants presented at least one sign or symptom of TMD. The most frequent tinnitus profile was characterized as high-pitched, continuous, and bilateral, with a mean pitch of 8.6 kHz and a mean loudness of 14.1 dB SL. Although the perceived annoyance was, on average, mild, it was observed that the higher the tinnitus pitch, the greater the THI score, suggesting a more bothersome perception of the symptom. In addition, asymmetric mouth-opening movement was the TMD sign most frequently identified in this group.

Another study^(14)^ demonstrated that patients with tinnitus associated with TMD exhibited significantly different characteristics when compared with individuals without TMD. One of the most relevant findings was that half of the patients with TMD reported the ability to modulate tinnitus through jaw or neck movements, whereas only 21% of patients without TMD reported the same phenomenon (p = 0.001), suggesting a somatosensory influence on tinnitus perception. When patients with TMD were subdivided into those whose primary complaint was tinnitus and those who primarily sought treatment for TMD, the subgroup with tinnitus as the chief complaint reported a greater impact on quality of life, as evidenced by higher THI scores. However, no additional significant differences were identified between these subgroups, indicating that the clinical parameters analyzed alone do not determine which symptom is perceived as more distressing by the patient.

### Risk of bias assessment

**Figure 2:**
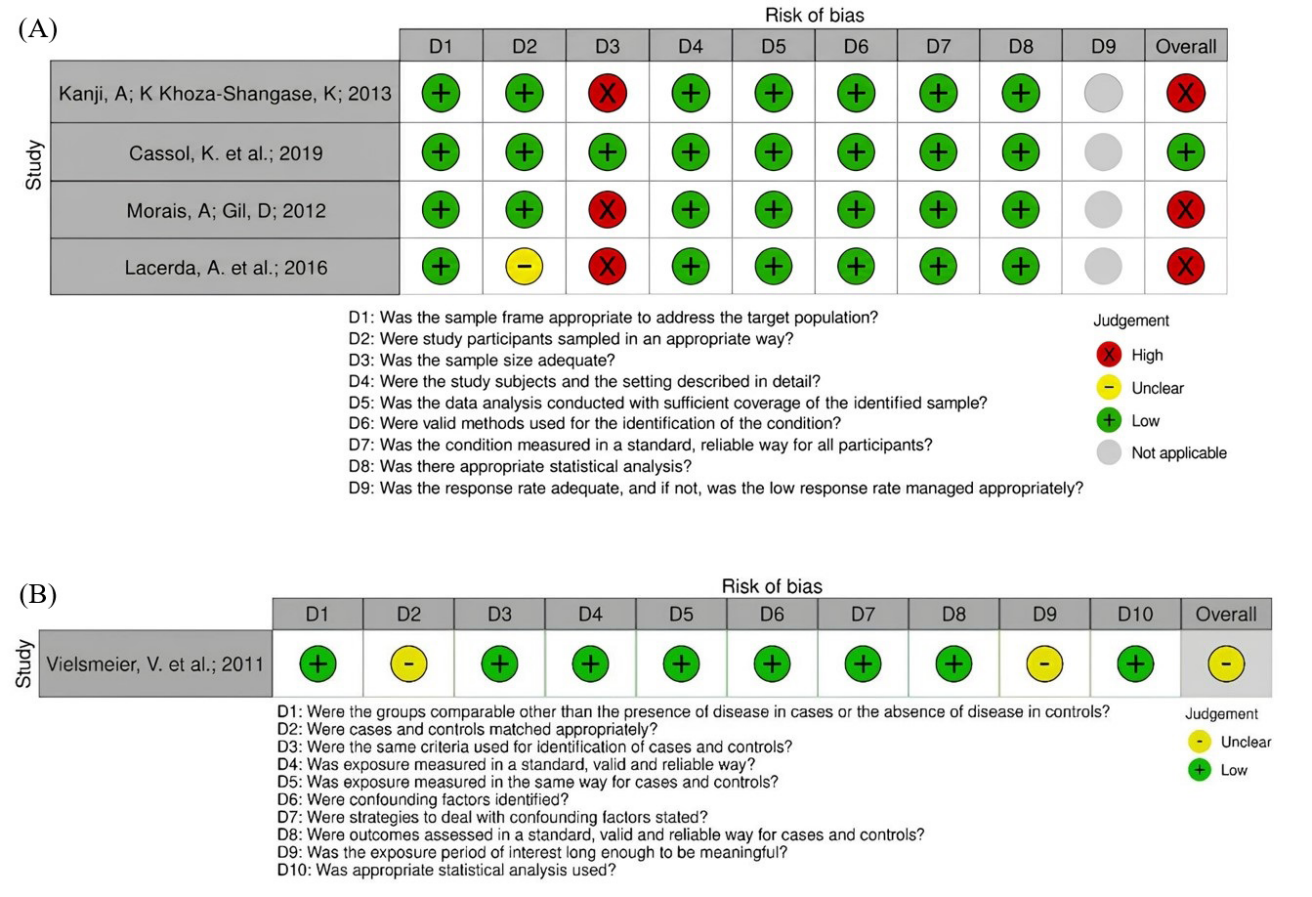
Risk of bias assessment of the studies included in the synthesis, evaluated using the JBI Critical Appraisal Tools. Green indicates low risk of bias, yellow indicates unclear risk of bias, and red indicates high risk of bias. (A) Cross-sectional studies; (B) Case-control studies

To assess the methodological quality of the selected studies, the Joanna Briggs Critical Appraisal tool^(15)^ was employed using the checklist appropriate for each study design. Cross-sectional studies were evaluated according to the nine domains of the *JBI Critical Appraisal Checklist for Studies Reporting Prevalence Data* (Table 2), whereas the case-control study was assessed using the ten specific domains of the *JBI Critical Appraisal Checklist for Case Control Studies* (Table 3). The Robvis software^(16)^ was used to generate the risk-of-bias figure (Figure 2).

**Table 2:** Risk of bias assessment of the included cross-sectional studies according to the JBI Critical Appraisal Tools

JBI Domain	Morais and Gil^(11)^	Lacerda et al.^(12)^	Cassol et al.^(10)^	Kanji and Khoza-Shangase^(13)^
D1 – Clearly defined inclusion criteria	Yes	Yes	Yes	Yes
D2 – Adequacy of the recruitment process	Yes	Unclear	Yes	Yes
D3 – Adequate sample size	No	No	Yes	Yes
D4 – Detailed description of participants and setting	No	No	Yes	Yes
D5 – Adequate sample coverage	Yes	Yes	Yes	Yes
D6 – Validity of the methods used to identify the condition	Yes	Yes	Yes	Yes
D7 – Valid and reliable outcome measurement	Yes	Yes	Yes	Yes
D8 – Appropriate statistical analysis	Yes	Yes	Yes	Yes
**Overall Judgment**	Moderate	Moderate-to-High	Low	Low

Caption: D = domain

**Table 3:** Risk of bias assessment of the included case-control study according to the JBI *Critical Appraisal Tools*

JBI Domain	Vielsmeier et al.^(14)^
D1 – Clearly defined case criteria	Yes
D2 – Representativeness of cases	Yes
D3 – Appropriate selection of controls	Yes
D4 – Comparability between cases and controls	Yes
D5 – Valid identification of the condition (cases)	Yes
D6 – Valid identification of the condition (controls)	Yes
D7 – Standardized outcome measurement	Yes
D8 – Management of confounding factors	unclear
D9 – Appropriate statistical analysis	Yes
Overall Judgment	low

Caption: D = domain

Regarding the cross-sectional studies, the overall analysis demonstrated low risk of bias in domains related to population definition and representativeness, diagnostic procedures, and the measurement of audiological and somatosensory variables. Most evaluated domains were classified as “low risk,” indicating adequate methodological consistency in these aspects.

Nevertheless, some domains presented relevant inconsistencies, particularly among cross-sectional studies. The domain related to sample size (D3) was judged as high risk in the studies by Morais and Gil^(11)^ and Lacerda et al.^(12)^, reflecting the small number of participants and the absence of sample size calculation or justification. Likewise, the domain related to the detailed description of the setting and participants (D4) was also classified as high risk in these studies because of the limited characterization of the sample and clinical context. In the study conducted by Lacerda et al.^(12)^, domain D2 was additionally judged as “unclear” due to the retrospective nature of data collection and the lack of clear information regarding the recruitment process. The study by Kanji and Khoza-Shangase^(13)^ received favorable ratings in nearly all domains, except for sample size (D3), which was classified as “unclear” due to the absence of formal justification, although without a clear indication of high risk. With respect to the case-control study, methodological performance was consistently satisfactory, with low risk identified across all domains except D2 and D9, both classified as “unclear” because of the limited description of matching procedures and exposure time. Although cases and controls were compared across several characteristics, no clear strategies were described to minimize selection bias or ensure representativeness between groups. Furthermore, although standardized criteria were used for identifying cases and controls through functional analysis of the masticatory system, the study did not provide a detailed description of matching procedures between groups, which may have introduced bias into the comparative analysis.

Overall, the critical appraisal findings indicated that, despite the heterogeneity observed among the included studies, no consistent patterns of bias were identified that would compromise the overall conclusions of this review. Nevertheless, domains presenting greater methodological fragility were carefully considered during interpretation of the findings.

A qualitative sensitivity analysis was also performed to determine whether the trends observed in the synthesis remained stable after exclusion of studies with a higher concentration of risk-of-bias judgments. Excluding the studies by Morais and Gil^(11)^ and Lacerda et al.^(12)^ did not substantially alter the overall patterns described in the review, suggesting that the preliminary conclusions were not exclusively dependent on these investigations. However, these findings should still be interpreted cautiously, given the limited number of studies and the methodological heterogeneity among them.

### Synthesis of results

Overall, the analyzed studies demonstrated that patients with TMD generally present high-frequency, continuous tinnitus^(11)^, predominantly unilateral in nature, although bilateral presentations may also occur, with loudness levels above 10 dB SL. Tinnitus may be modulated by somatosensory stimuli and appears to exert a mild to moderate impact on quality of life^(13)^. The occurrence of tinnitus in individuals with normal hearing and signs of TMD reinforces the importance of multidisciplinary assessment and integrated management involving speech-language pathology, dentistry, and other healthcare professionals.

The quantitative synthesis of the five included studies revealed a predominance of high-pitched tinnitus, with an approximate mean pitch of 6,790 Hz (ranging from 3,762 to 8,600 Hz) among the studies that performed acuphenometry. Mean loudness ranged between 14.1- and 33.5-dB SL, with a central value of approximately 19.6 dB SL. Regarding laterality, unilateral tinnitus predominated among patients with TMD, particularly in the study by Cassol et al.^(10)^, in which 98% of cases demonstrated correspondence between the side affected by TMD and the side of tinnitus perception. Among studies reporting bilateral distribution, an approximate prevalence of 60% bilateral tinnitus was observed in Morais and Gil^(11)^, whereas Lacerda et al.^(12)^ reported right-sided predominance (42%).

Concerning somatosensory modulation, the case-control study conducted by Vielsmeier et al.^(14)^ demonstrated that 66% of individuals with TMD experienced changes in tinnitus perception during mandibular or cervical movements, reinforcing the role of somatosensory mechanisms in tinnitus pathophysiology.

The analyzed studies presented important methodological strengths, including the use of validated instruments such as the THI, acuphenometry, and detailed methodological approaches^(10-13)^. However, relevant limitations were also identified, particularly regarding sample size and participant selection strategies. Therefore, although the included studies provide valuable contributions to understanding the relationship between tinnitus and TMD, further primary studies with larger sample sizes and clearly defined sampling methods are still required. Such improvements are essential to increase the reliability and applicability of the available evidence.

**Figure 3:**
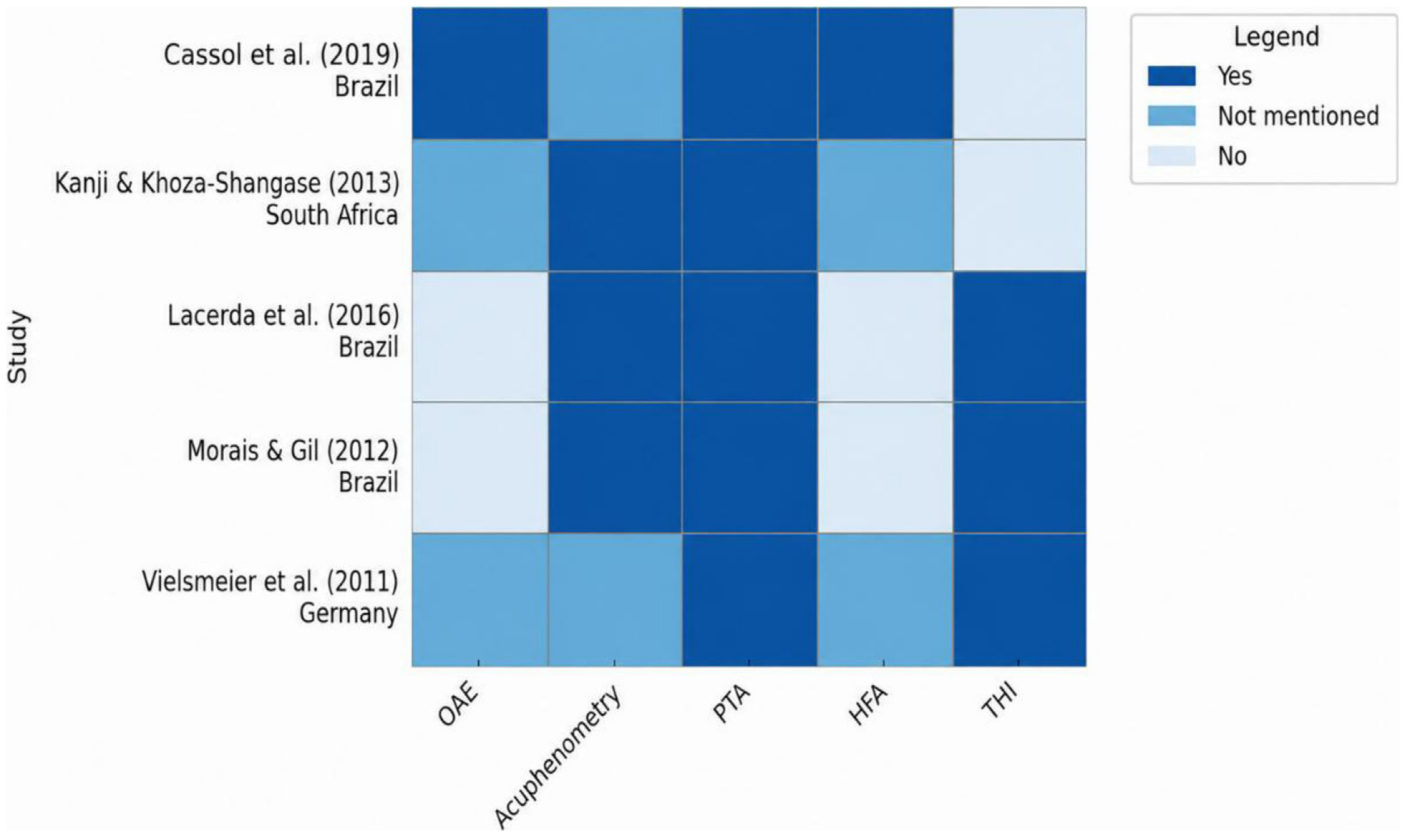
*Heatmap:* Audiological assessments and instruments used

Legend: *Heatmap* of the audiological procedures reported in the included studies. The shades of blue represent the presence, absence, or lack of mention of each procedure in each study: dark blue (yes): the procedure was clearly described and performed in the study; medium blue (not mentioned): the study did not mention the procedure, making it impossible to confirm whether it was performed; light blue (no): the study explicitly stated that the procedure was not performed. The mapped procedures were: Otoacoustic Emissions (OAE), Acuphenometry, Conventional Pure-Tone Audiometry (PTA), High-Frequency Audiometry (HFA), and Tinnitus Handicap Inventory (THI). The figure allows visualization of which assessments were conducted in each study and identification of patterns in the use of audiological instruments

Furthermore, among the five studies included in the synthesis, only one study^(10)^, for example, performed high-frequency audiometry (HFA). In the heatmap presented in Figure 3 below, each study was categorized according to three methodological conditions related to audiological procedures: “yes” (procedure explicitly performed), “no” (procedure explicitly not performed), and “not mentioned” (absence of information regarding the procedure in the study). These categories were converted into graduated shades of blue, in which darker colors indicated greater methodological completeness and clarity (explicitly reported items), whereas lighter shades represented information gaps. This color-coding strategy enabled visualization of the heterogeneity across studies, both regarding the presence or absence of specific procedures and the degree of transparency in their reporting.

## DISCUSSION

The present systematic review aimed to identify and describe the characteristics of tinnitus associated with temporomandibular disorder (TMD), considering the already established relationship between these two conditions^(17,18)^.

The findings reported by Ren and Isberg^(19)^ demonstrated that, in all evaluated patients, unilateral tinnitus occurred on the same side as the temporomandibular joint (TMJ) disc displacement, corroborating the postulation proposed by Costen^(20)^, who suggested that mechanical alterations of the TMJ could compromise the auriculotemporal nerve, a sensory branch of the trigeminal nerve that innervates both the joint capsule and structures of the auditory canal, in addition to modifying intratympanic pressure and consequently generating ipsilateral otologic symptoms. Review studies such as those conducted by Ralli et al.^(21)^ and Michiels et al.^(22)^ have emphasized that trigeminal and cervical somatosensory pathways converge early within the auditory system, modulating the excitability of the dorsal cochlear nucleus (DCN) and influencing the integration between auditory and somatic afferents. These neurophysiological models, together with the observation that somatic lesions may trigger ipsilateral tinnitus, support the hypothesis that somatosensory mechanisms may contribute to tinnitus perception on the same side as dysfunction.

Nevertheless, the strength of this association remains limited by the small sample sizes and the heterogeneity of the diagnostic criteria employed. Therefore, studies with greater statistical power and standardized assessment of tinnitus laterality associated with TMD are still required.

Following data analysis, tinnitus associated with TMD was shown to be generally characterized by higher pitch values, typically around 6 kHz. In addition, tinnitus loudness was found to be moderate and commonly less intense than in patients whose tinnitus symptoms originated from factors other than TMD^(23)^.

Regarding the findings of the present review concerning high-frequency pitch, moderate loudness, and somatosensory tinnitus, the literature demonstrates an early convergence of trigeminal and cervical somatosensory afferents within the dorsal cochlear nucleus (DCN), modulating its excitability and triggering maladaptive plasticity in central auditory circuits, even in individuals with normal or only slightly altered peripheral hearing^(21,22,24)^. Experimental evidence indicates that, in the presence of persistent somatic irritation or minimal auditory deprivation, the DCN exhibits increased firing rates, greater neuronal synchrony, and enhanced excitatory influence arising from somatosensory pathways^(25)^, preferentially affecting high-frequency tonotopic regions. Furthermore, because these patients frequently maintain relatively preserved peripheral hearing, tinnitus appears to result more from somatosensory modulation than from cochlear damage itself. In such cases, dysregulated neural activity may be sufficient to generate tinnitus perception without marked central amplification, which may explain the moderate loudness observed^(24)^.

Although the characteristics identified in the present review may suggest a specific tinnitus profile associated with TMD, it is important to emphasize that other clinical conditions, such as hidden hearing loss and noise-induced hearing loss (NIHL), may also present tinnitus with pitch values around 6 kHz^(26,27)^. This overlap of characteristics across different pathologies makes it difficult to identify a unique or distinctive tinnitus subtype exclusively associated with TMD. Therefore, establishing a specific acoustic profile or a single tinnitus characteristic that could be considered a clear and exclusive indicator of TMD remains inconclusive^(10,13)^.

The differentiation between tinnitus associated with TMD and noise-induced tinnitus, however, has important clinical implications. Tinnitus related to TMD tends to occur in individuals with normal or only mildly altered peripheral hearing and may frequently be accompanied by somatic modulation and/or orofacial pain^(22)^. Conversely, large population-based studies on occupational noise exposure have demonstrated that noise-induced tinnitus is more prevalent among chronically exposed workers and is strongly associated with sensorineural hearing loss, particularly at frequencies between 3 and 6 kHz^(28)^. This contrast reinforces the importance of considering the audiological profile, occupational history, and presence of somatic sensitivity when classifying tinnitus subtypes in clinical practice.

In younger patients with tinnitus associated with TMD, the symptom may be relieved through movements of the jaw, head, or neck. In such cases, classical tinnitus risk factors, including advanced age, male sex, and hearing loss, appear to be less relevant. Based on these findings, treatment of TMD may represent a causally oriented therapeutic approach for tinnitus^(14,19)^. These observations further highlight the importance of careful assessment and a multidisciplinary approach in tinnitus management, including the involvement of dental professionals^(14)^, considering that this condition may significantly impact both diagnostic processes and therapeutic management.

The results also indicated that the presence of auditory alterations in patients with TMD is clinically significant. Therefore, these patients should be informed about the possible causes of this condition. In addition, audiological follow-up should be recommended for individuals with TMD regardless of the presence of tinnitus, in order to monitor symptom progression and enable appropriate interventions^(19)^.

The observation that half of the studies selected for full-text analysis were Brazilian investigations^(10-12)^ highlights the relevance of this research topic within the national context. Furthermore, it demonstrates the advancement of Brazilian audiological research within the international scientific scenario. This finding suggests methodological and cultural adequacy in tinnitus research and reflects a tendency, particularly within the Brazilian context, to prioritize the investigation of psychoacoustic aspects.

Although these studies provide important contributions, especially regarding the psychoacoustic characterization of tinnitus associated with TMD, some limitations must be acknowledged. Among these, the most notable are the small sample sizes^(13)^, and the adoption of restricted assessment protocols. In several cases, investigations relied exclusively on subjective impact measures, such as the Tinnitus Handicap Inventory (THI), without incorporating more comprehensive audiological evaluations, including acuphenometry, specific somatosensory modulation tests, and high-frequency audiometry. These methodological limitations reduce comparability among studies and limit the ability to establish more robust conclusions regarding the psychoacoustic profile of tinnitus in patients with TMD.

Additionally, heterogeneity was observed in the diagnostic criteria for TMD, in the standardization of acuphenometry procedures, and in the instruments used to quantify tinnitus-related distress, such as the THI. Incomplete reporting of complementary audiological procedures also hindered comparisons among studies and limited inferences regarding the relationship between cochlear integrity and tinnitus characteristics.

Although three studies presented low risk of bias according to the JBI assessment tools, two studies demonstrated moderate risk of bias, particularly in domains related to external validity and control of confounding factors. Therefore, although the findings were generally consistent across studies, the overall certainty of evidence remains limited, reinforcing the need for longitudinal studies with more robust methodological designs, standardized diagnostic criteria, and greater control of audiological and somatosensory variables.

The available evidence reinforces the importance of standardized audiological and psychoacoustic protocols for patients with TMD, including pitch matching and loudness matching procedures. Improved understanding of these parameters may contribute to differentiation of somatosensory tinnitus subtypes and facilitate the development of integrated therapeutic approaches involving orofacial myofunctional therapy, auditory rehabilitation, physiotherapy, and dental management.

## CONCLUSION

The findings of the present systematic review demonstrated that tinnitus in patients diagnosed with TMD is predominantly continuous, generally unilateral and associated with the side affected by the dysfunction, with high pitch values and mean frequencies ranging between 3,750 Hz and 8,600 Hz, as well as mean loudness values between 14 dB SL and 25 dB SL. Tinnitus may occur either in the presence or absence of hearing loss, and even in cases with normal auditory thresholds, audiological monitoring is recommended. A moderate impact on quality of life was identified, with associations observed between symptom duration, pitch, and scores obtained through specific quality-of-life assessment instruments. An especially important finding was that patients with TMD demonstrated modulation of tinnitus during mandibular and cervical movements, distinguishing them from cases in which tinnitus was not associated with the investigated dysfunction.

## Appendix A Search strategies

**Table:**

**DATABASE**	**SEARCH STRATEGY**
PUBMED	("*temporomandibular joint disorders*" [MeSH Terms] OR "*Temporomandibular Disorders*" OR "*Temporomandibular Joint Disorder*" OR "*Temporomandibular Joint Diseases*" OR "*Temporomandibular Disorders*") AND ("*tinnitus*" [MeSH Terms] OR tinnitus) AND (*pitch* [All Fields] OR *loudness* [All Fields] OR *characteristics* [All Fields])
EMBASE	('*temporomandibular joint disorders*' OR '*Temporomandibular Disorders*' OR '*Temporomandibular Joint Disorder*' OR '*Temporomandibular Joint Diseases*' OR '*Temporomandibular Disorders*') AND ('*tinnitus*' or*tinnitus*) AND (*pitch* OR *loudness* OR *characteristics*)
LILACS	(MH:"*Temporomandibular Joint Dysfunction Syndrome*" OR "*Temporomandibular Joint Dysfunction Syndrome*" OR "Síndrome da Disfunção da Articulação Temporomandibular" OR "*Síndrome de la Disfunción de Articulación Temporomandibular*" OR "*Síndrome Miofascial de Disfunção Dolorosa Temporomandibular*" OR "Síndrome da ATM" OR "Síndrome da Articulação Temporomandibular" OR MH: C05.500.607.221.897.897 OR MH: C05.550.905.905 OR MH: C05.651.243.897.897 OR MH: C05.651.550.905 OR MH: C07.320.610.291.897.897 OR MH: C07.678.949) AND (MH:"*tinnitus*" OR "*tinnitus*" OR "zumbido" OR "*acúfeno*" OR "*tinido*" OR "zumbido pulsátil" OR "*zumbidos*" OR "*zunido*" OR MH:C09.218.458.670 OR MH:C10.597.751.418.670 OR MH: C23.888.592.763.393.670)
Web Of Science	("*temporomandibular joint disorders*" OR "*Temporomandibular Disorders*" OR "*Temporomandibular Joint Disorder*" OR "*Temporomandibular Joint Diseases*" OR "*Temporomandibular Disorders*") AND ("*tinnitus*") AND (*pitch* OR *loudness* OR *characteristics*)
Scopus	("*temporomandibular joint disorders*" OR "*Temporomandibular Disorders*" OR "*Temporomandibular Joint Disorder*" OR "*Temporomandibular Joint Diseases*" OR "*Temporomandibular Disorders*") AND ("*tinnitus*" OR *tinnitus*) AND (*pitch* OR *loudness* OR *characteristics*)
Google Scholar	"*temporomandibular joint disorders*" OR "*Temporomandibular Disorders*" OR "*Temporomandibular Joint Disorder*" OR "*Temporomandibular Joint Diseases*" OR "*Temporomandibular Disorders*" AND "*tinnitus*" AND *pitch* OR *loudness* OR *characteristics*filetype:pdf

## Appendix B Excluded studies and respective reasons for exclusion (n = 13)

**Table:**

**Author**	**Reason for Exclusion**
Pavaci et al., 2019	1
Mazzetto et al., 2013	2
Bousema et al., 2018	3
Michiels et al., 2018	2
Pozuelo-Pinilla et al., 2010	3
Michiels, 2020	3
Mahmoudian et al., 2022	4
Giordani, 1993	2
Clauser et al., 2011	2
Upton and Wijeyesakere, 2004	2
Sanchez et al., 2007	1
Vielsmeier et al., 2012	1
van Oijen, 2022	1

1 Studies that did not investigate the outcome of interest

2 The study did not report psychoacoustic tinnitus data

3 Review study

4 Case series study
