# Supplementary material for: Psychoacoustic characteristics of tinnitus in temporomandibular disorders: a systematic review
Source: Codas. 2026 Jul 27;38(5):e20250343. doi: 10.1590/2317-1782/e20250343en (PMC13440683; doi:10.1590/2317-1782/e20250343en)
Supplement: Translation

# Translation: As características psicoacústicas do zumbido nas disfunções temporomandibulares: uma revisão sistemática

**DOI:** 10.1590/2317-1782/e20250343pt

**Authors:** Taís de Azevedo Picinini, Ingrid Eismann Moraes, Karen Dantur Batista Chaves, Alexandre Hundertmarck Lessa, Adriana Laybauer Silveira Unchalo, Adriane Ribeiro Teixeira

**Affiliations:** 1 Programa de Pós-graduação em Saúde da Comunicação Humana, Universidade Tuiuti do Paraná – UTP - Curitiba, PR, Brasil.; 2 Curso de Fonoaudiologia, Universidade Federal do Rio Grande do Sul – UFRGS - Porto Alegre (RS), Brasil.; 3 Departamento de Odontologia Conservadora, Faculdade de Odontologia, Universidade Federal do Rio Grande do Sul – UFRGS - Porto Alegre (RS), Brasil.; 4 Departamento de Saúde e Comunicação Humana, Instituto de Psicologia, Serviço Social, Saúde e Comunicação Humana, Universidade Federal do Rio Grande do Sul – UFRGS - Porto Alegre (RS), Brasil.; 5 Serviço de Fonoaudiologia, Hospital de Clínicas de Porto Alegre – HCPA - Porto Alegre (RS), Brasil.

**Keywords:** Audição, Audiologia, Psicoacústica, Síndrome da Disfunção da Articulação Temporomandibular, Zumbido

## Abstract

**Objetivo:**

Investigar características psicoacústicas do zumbido em pacientes diagnosticados com disfunção temporomandibular (DTM) e descrever os achados obtidos na avaliação audiológica básica.

**Estratégia de pesquisa:**

Foi realizada revisão sistemática de acordo com as diretrizes PRISMA, com protocolo registrado no PROSPERO (CRD42025649038). As buscas abrangeram as bases Embase, LILACS, PubMed/MEDLINE, Scopus, Web of Science e Google Scholar, utilizando descritores validados em MeSH e DeCS.

**Critérios de seleção:**

Incluídos estudos descritivos, observacionais, transversais, caso-controle ou longitudinais que avaliaram pacientes com DTM e zumbido, relatando características psicoacústicas como pitch e loudness. Foram excluídos estudos com animais, casos isolados, séries de casos, revisões, cartas, editoriais e artigos sem dados originais.

**Análise dos dados:**

Dois revisores realizaram a triagem de títulos, resumos e textos completos, com consulta a um terceiro revisor, nas divergências. O risco de viés foi avaliado com as ferramentas JBI Critical Appraisal Tools. Os dados foram extraídos, organizados e sintetizados qualitativamente.

**Resultados:**

Inicialmente, 271 artigos foram identificados; após exclusões e análises, cinco estudos foram incluídos na síntese. Os resultados indicam que o zumbido em pacientes com DTM é predominantemente unilateral, contínuo, de pitch elevado e loudness moderada. Uma parte dos pacientes consegue modular o zumbido por movimentos mandibulares ou cervicais, sugerindo influência somatossensorial. O impacto na qualidade de vida varia de leve a moderado, mesmo em indivíduos com audição normal.

**Conclusão:**

O zumbido associado à DTM apresenta características psicoacústicas relativamente consistentes, mas há necessidade de mais estudos primários com amostras maiores e delineamento robusto para confirmar padrões específicos e aprimorar intervenções multidisciplinares.

## INTRODUÇÃO

O zumbido é uma sensação auditiva não associada a uma fonte externa e geralmente causa grande incômodo, resultando em perda na qualidade de vida do indivíduo. É um sintoma que pode ser causado por diversas condições e patologias, sendo a disfunção temporomandibular (DTM) uma delas.^(1)^ Geralmente é percebido e relatado como um chiado ou assobio nas orelhas e/ou na cabeça. Embora seja comumente referido como “zumbido nos ouvidos”, essa condição pode se manifestar de formas diferentes, incluindo um zumbido propriamente dito, mas também sons como “swoosh” e cliques^(2,3)^.

Em relação ao tempo de duração da sensação, pode ser caracterizado como agudo quando dura por um breve período, como após um show de alta intensidade sonora. Também pode ser considerado uma condição de saúde crônica, quando o indivíduo percebe o zumbido de forma incessante, ou, ainda, pode aparecer de forma intermitente. Ademais, alguns pacientes relatam que se trata de condição extremamente incapacitante, enquanto outros referem que a sensação é quase imperceptível^(2-4)^.

O zumbido pode ser desencadeado por diversas condições, como perda auditiva induzida por ruído, presbiacusia, uso de medicamentos ototóxicos, distúrbios metabólicos e alterações musculoesqueléticas, incluindo as disfunções temporomandibulares (DTM), que têm sido progressivamente reconhecidas como um fator associado à gênese e modulação do sintoma.

A DTM é uma condição que engloba um conjunto variado de problemas musculoesqueléticos e neuromusculares relacionados à articulação temporomandibular (ATM), aos músculos da mastigação e estruturas adjacentes. Seus sinais e sintomas podem incluir estalos, alterações na função da mandíbula e dores. ^(5)^ De acordo com a Academia Americana de Dor Orofacial, a DTM é definida como um grupo de distúrbios que envolvem os músculos mastigatórios, a articulação temporomandibular (ATM) e as estruturas associadas. ^(6)^

Embora a relação entre DTM e zumbido seja reconhecida, a caracterização psicoacústica específica desse sintoma, em termos de pitch e loudness, e suas correlações com as características audiológicas obtidas em exames como a audiometria tonal liminar e a imitanciometria permanecem inconsistentes entre os estudos. Até o momento, não há revisão sistemática que integre tais achados. Essa lacuna justifica a realização da presente revisão, que tem como objetivo sintetizar as evidências disponíveis e descrever as características psicoacústicas e audiológicas do zumbido em indivíduos com DTM, buscando identificar padrões clínicos que orientem futuras investigações e subsidiem estratégias terapêuticas mais direcionadas.

## MÉTODO

### Estratégias de busca e critérios de elegibilidade

A pergunta de pesquisa foi: “Quais são as características psicoacústicas do zumbido em pacientes com DTM?”. A estratégia com acrônimo PECOS (população, exposição, comparação desfecho/outcome e *studies design*) adotada foi a seguinte:

P (população): pacientes com DTM;

E (exposição): presença de zumbido;

C (comparação): qualquer outro grupo de indivíduos com zumbido e sem DTM;

O (desfecho): características do zumbido;

S (desenhos de estudo): estudos descritivos, observacionais, transversais, caso-controle ou longitudinais.

A pesquisa foi conduzida de acordo com as diretrizes do *Preferred Reporting Items for Systematic Reviews and Meta-Analyses* (PRISMA) ^(7)^ seguindo as etapas recomendadas no *Cochrane Handbook for Systematic Reviews* ^(8)^ que incluem: formulação da pergunta de pesquisa, identificação e seleção dos estudos, avaliação crítica da qualidade metodológica, extração, análise e apresentação dos dados, interpretação dos resultados e, por fim, aprimoramento e atualização da revisão. O protocolo da presente revisão foi registrado na plataforma PROSPERO (CRD42025649038).

As bases de dados consultadas foram Embase, Literatura Latino-Americana e do Caribe em Ciências da Saúde (LILACS), PubMed/MEDLINE, Scopus, *Web of Science* e Google Scholar, para literatura cinzenta.

As buscas às bases de dados foram realizadas em setembro de 2024 e a última atualização foi em 14 de outubro de 2024, nas seguintes bases: Embase, Literatura Latino-Americana e do Caribe em Ciências da Saúde (LILACS), PubMed/MEDLINE, Scopus, Web of Science e Google Scholar. Todas as estratégias de busca foram desenvolvidas e revisadas por dois pesquisadores experientes. Os descritores utilizados foram verificados nos vocabulários controlados *Medical Subject Headings* (MeSH) e Descritores em Ciências da Saúde (DeCS), acessados por meio do portal de periódicos da CAPES^(9)^ e, a partir deles, foram elaboradas estratégias de busca para cada base de dados, combinando os descritores selecionados, conforme detalhado no Apêndice A. Não foram aplicados filtros por tipo de estudo, idioma ou data de publicação. Não houve contato com autores para obtenção de dados adicionais.

A busca no Google Scholar foi realizada para identificar literatura cinzenta relevante. Não foram aplicadas restrições quanto ao idioma. A configuração da página foi ajustada para exibir 20 resultados por página, sendo considerados os primeiros 100 resultados (cinco páginas), ordenados por relevância. Foi utilizado o comando ‘filetype:pdf’ para restringir a busca a arquivos em formato *Portable Document Format* (PDF) com texto completo disponível. A triagem dos resultados foi realizada com base na leitura dos títulos e resumos, sendo incluídos apenas os estudos que atendiam aos critérios de elegibilidade definidos.

Embora a síntese dos achados tenha sido descritiva, este estudo manteve o delineamento de revisão sistemática por atender integralmente às etapas preconizadas pelo PRISMA, incluindo busca estruturada, critérios de elegibilidade definidos e avaliação crítica da qualidade metodológica dos estudos.

## CRITÉRIOS DE SELEÇÃO

Os estudos foram selecionados e revisados de forma independente por dois avaliadores, conforme os critérios de inclusão e exclusão previamente definidos. O coeficiente de concordância Kappa entre os revisores foi superior a 0,7, indicando alto nível de concordância.

### Critérios de inclusão

Foram incluídos na revisão estudos publicados em português, inglês ou espanhol, sem restrição de data. Foram considerados elegíveis estudos que envolveram pacientes de qualquer faixa etária submetidos a algum tipo de avaliação auditiva e que apresentavam zumbido associado à DTM. Os estudos deveriam relatar dados psicoacústicos do zumbido, como *pitch*, *loudness*, ou outras características descritivas. Foram aceitos estudos observacionais, descritivos, transversais ou longitudinais que abordassem o desfecho de interesse desta revisão.

### Critérios de exclusão

Foram estabelecidos como critério de exclusão os artigos que não contavam com as características psicoacústicas do zumbido na DTM, bem como estudo de casos, séries de casos, revisões de literatura, cartas ao editor, editoriais e artigos de opinião que não apresentam dados originais e estudos com uso de modelo com animais.

## SELEÇÃO DOS ESTUDOS E ANÁLISE DOS DADOS

Os dados foram extraídos com base nas estratégias de busca definidas para cada base de dados, exportados no formato RIS e organizados no gerenciador de referências Zotero. Em seguida foram importados para a plataforma de triagem Rayyan onde a Fase 1 foi iniciada. Nessa etapa, dois revisores realizaram, de forma independente, a leitura dos títulos e resumos de todos os estudos recuperados, excluindo aqueles que não atendiam aos critérios estabelecidos. Em casos de dúvida quanto à elegibilidade, procedeu-se à leitura integral do artigo. Na Fase 2, os mesmos revisores conduziram a leitura na íntegra dos estudos potencialmente elegíveis. Quando houve divergência entre os avaliadores, um terceiro revisor atuou como árbitro.

### Extração e síntese de dados

A extração dos dados dos estudos selecionados para a síntese foi realizada de forma independente pelos dois revisores. Para cada estudo incluído foram coletadas as seguintes informações: autor, ano de publicação, país, delineamento do estudo, tamanho e características da amostra, instrumentos e métodos utilizados para avaliação auditiva e para mensuração das características psicoacústicas do zumbido (*pitch, loudness*, tipo de estímulo, lateralidade, entre outros), principais resultados e conclusões.

As divergências na extração foram discutidas até se alcançar consenso, com a mediação de um terceiro revisor quando necessário. A síntese dos achados foi conduzida de forma descritiva e qualitativa, considerando as variáveis mais frequentemente relatadas nos estudos. Os resultados foram organizados em tabelas de extração e analisados quanto as tendências observadas nas medidas psicoacústicas e suas possíveis relações com os achados audiológicos.

## RESULTADOS

### Seleção de fontes de evidência

Inicialmente, foram encontrados 271 artigos nas bases de dados eletrônicas. Desses, 137 eram duplicatas, das quais 49 foram resolvidas e 88 excluídas. Após a exclusão das duplicatas, procedeu-se à análise dos títulos e resumos de 183 artigos de acordo com os critérios de inclusão e exclusão, resultando na exclusão de 153 artigos. Dez artigos ficaram em conflito e foram encaminhados ao juiz para desempate. Destes, 18 artigos foram selecionados para a leitura na íntegra, sendo selecionados cinco artigos para a análise e síntese (Figura 1). No Apêndice B são explicadas as razões de exclusão de cada estudo não incluído na presente revisão.

**Figura 1:** Diagrama de fluxo PRISMA da seleção dos artigos incluídos na revisão sistemática

Fonte*:* Page et al.^(7)^. Para mais informações, visite o site PRISMA^(29)^

### Resultados de fontes individuais de evidência

Os estudos incluídos foram produzidos entre os anos de 2011 e 2019, incluindo produções brasileiras^(10-12)^ e estudos da África do Sul e da Alemanha^(13,14)^. A Tabela 1, a seguir, resume os dados extraídos dos estudos, incluindo autor, ano de publicação, país de origem, tipo de estudo, amostra, idade e sexo dos participantes, objetivos, procedimentos de avaliação, características do zumbido avaliadas, principais resultados e conclusão.

**Tabela 1:** Extração de dados dos estudos incluídos (N = 5)

**Autor e Ano (País/ Tipo de estudo)**	**Amostra, Idade e sexo**	**Objetivo**	**Procedimentos de avaliação**	**Características do zumbido avaliadas**	**Resultados**	**Conclusão**
Cassol et al.^(10)^, 2019 (Brasil)/ Observacional descritivo, transversal)	53 pacientes: 7 do sexo masculino e 46 do sexo feminino; idades entre 22 a 69 anos, com média de 41 anos.	Investigar a saúde auditiva de portadores de zumbido associado à DTM na etapa pré-tratamento odontológico	Audiometria tonal limiar convencional (AT-AC)	Lateralidade do zumbido	O zumbido apresentou forte relação com o lado da DTM ocorrendo em 52 de um total de 53 participantes analisados (98%). Quando foi observado DTM bilateral, o zumbido também se mostrou bilateral e mais intenso do lado em que a DTM é mais intensa.	Todos os pacientes portadores de DTM, associados ou não à presença de zumbido, devem ser indicados ao monitoramento audiológico.
			Logoaudiometria		O zumbido unilateral foi relatado pela maioria dos indivíduos, sendo com presença de plenitude auricular (77%) e sem otalgia (53%).	
			Medidas de Imitância acústica	Plenitude auricular e otalgia	A maioria dos sujeitos apresentou limiares auditivos normais bilateralmente (OD= 43%; OE= 50%) seguido de perda moderada (OD= 30%; OE= 25%) e pior desempenho na AAF em relação à AT-CV.	
			Audiometria tonal de altas frequências (AAF)			
			Emissões otoacústicas (EOA)		50% dos indivíduos apresentaram ausência de emissões otoacústicas evocadas por estímulo transiente (EOAET), mesmo com audiometria convencional normal.	
Kanji e Khoza-Shangase^(13)^, 2013 (África do Sul)/ Este estudo piloto exploratório, comparativo, não experimental	20 participantes:	Identificar os sinais e sintomas clínicos do zumbido em pacientes com DTM.	Questionário (adaptado do questionário de zumbido da *Carolina Ear and Hearing Clinic* (2005)) relacionado à percepção do seu zumbido e impacto que o zumbido causa em seu estilo de vida.	*Pitch, loudness,* início do zumbido, sua duração e localização	No grupo sem DTM (grupo B), a maioria (8 de 10) relatou zumbido de frequência e duração constantes, enquanto no grupo com DTM (grupo A), 3 de 10 relataram essa constância. Todos os participantes do grupo A descreveram o zumbido como um som único, enquanto três do grupo B relataram múltiplos sons. Os sons mais relatados no grupo A foram zumbido do tipo “*ringing*” e “*whistling*”, e no grupo B dos tipos “*ringing*” e “*buzzing*”.	As características do zumbido em pacientes com DTM mostraram-se diferentes daquelas observadas em pacientes sem DTM.
	Grupo A com DTM: 10 participantes (1 do sexo masculino e 9 do sexo feminino)				Quanto à *loudness*, não houve diferença estatisticamente significativa entre os grupos, entretanto o grupo com DTM apresentou intensidade média mais baixa (25,4 dB), comparada ao grupo B (33,5 dB). Quanto ao *pitch*, oito participantes do grupo A associaram seu zumbido a 6kHz (6 com tom puro e 2 com ruído estreito). No grupo sem DTM, metade pareou com 8kHz, três com 1kHz, um com 3kHz e um com ruído de 250 Hz.	O zumbido pode ocorrer em pacientes com DTM e ser de alta frequência, o que destaca a importância de uma avaliação cuidadosa em pacientes com zumbido, já que isso pode ter implicações no diagnóstico e no manejo clínico.
			Audiometria tonal limiar convencional (AT-AC)			
	Grupo B sem DTM: 10 participantes (5 do sexo masculino e 5 do sexo feminino)		Logoaudiometria			
			Medidas de Imitância acústica			
			Medições psicoacústicas do zumbido			
Lacerda et al.^(12)^, 2016 (Brasil) / Observacional,descritivo, transversal	19 indivíduos com DTM, com predomínio do sexo feminino na amostra (89,47%).	Avaliar o impacto do zumbido na qualidade de vida de pacientes com DTM	Audiometria tonal liminar, Acufenometria e questionário THI brasileiro	Tempo desde o início do sintoma, descrição do som, localização, tipo, duração e frequência.	63,12% zumbido <cinco anos, e 15,79% relataram um zumbido com sons semelhantes a assobio, chuva e/ou grilo.	
					Quanto à lateralidade, houve predomínio da orelha direita (42,11%) e 52,63% referiram que o zumbido é contínuo.	Observou-se uma correlação significativa entre a escala do THI, o tempo de duração do zumbido e sua frequência de ocorrência.
	A idade média foi de 53,5 anos (de 26 a 72 anos).				Na audiometria, os limiares audiométricos médios estiveram acima de 25 dB NA nas frequências de 3000, 4000, 6000 e 8000 Hz bilateralmente.	
					Na acufenometria, a média da intensidade do zumbido foi de 21 dB NS na orelha direita e 17,85 dB NS na orelha esquerda, e a média da frequência do zumbido foi de 3775 Hz na orelha direita e 3750 Hz na orelha esquerda.	O zumbido relatado por pacientes com DTM causou impacto moderado na qualidade de vida e pode ser percebido mesmo na presença de ruído de fundo, mesmo que as atividades diárias ainda possam ser realizadas.
					A pontuação total no THI foi de 37,8 pontos.	
Vielsmeier et al.^(14)^, 2011 (Alemanha) / Caso-controle	30 pacientes com diagnóstico confirmado de DTM e zumbido com um grupo de 61 pacientes com zumbido mas sem queixas subjetivas de DTM	Investigar diferenças nas características clínicas e demográficas entre os grupos.	Avaliação otorrinolaringológica, incluindo otoscopia e audiometria tonal liminar.	Idade de início, tipo de início (gradual ou abrupto), tipo de zumbido (pulsátil ou não pulsátil), intensidade, percepção/	Pacientes com zumbido e DTM apresentaram melhor função auditiva (P < 0,0005), menor idade (P = 0,001) e idade mais precoce de início do zumbido (P = 0,002), além de serem mais frequentemente do sexo feminino (P = 0,003). Esses pacientes também relataram menor intensidade subjetiva do zumbido (P = 0,01) e maior capacidade de modulação do zumbido por movimentos da mandíbula ou pescoço (P = 0,001).	Fatores de risco clássicos para o zumbido (idade, sexo masculino e perda auditiva) são menos relevantes em pacientes com zumbido e DTM, o que sugere um papel causal da patologia da ATM na gênese e manutenção do zumbido. Com base nesse achado, o tratamento da DTM pode representar uma estratégia terapêutica orientada para o zumbido.
	Idade média da amostra total: 49,1 anos		Questionário de Histórico de Caso de Zumbido e a versão alemã do Inventário de Incapacidade pelo Zumbido (THI).	consciência do zumbido, mascaramento modulação somatossensorial, hiperacusia e incapacidade relacionada ao zumbido avaliada pelo THI.	Além disso, a intensidade percebida do zumbido foi maior entre os pacientes sem DTM (P = 0,01; diferença média: 13,1; IC 95%: 3,1–23,1).	
	56 homens (61,5%), 35 mulheres (38,5%).				Os demais fatores avaliados não apresentaram diferenças significativas entre os grupos.	
				
Morais e Gil^(11)^, 2012 (Brasil) / Observacional, transversal, descritivo	Caracterizar o zumbido em indivíduos com audição normal e investigar a relação com a DTM	20 adultos de ambos os sexos (70% sexo feminino) com queixa de zumbido e limiares auditivos dentro da normalidade.	História audiológica e otológica, acufenometria e aplicação de um *checklist* de sinais e sintomas de DTM, o qual foi elaborado pela pesquisadora a partir de dados da literatura consulta com profissionais da área e questionário Tinnitus Handicap Inventory (THI).	Investigação da lateralidade, tipo e duração do zumbido e identificação do *pitch* e *loudness*	A maioria relatou zumbido de *pitch* agudo (75%), contínuo (90%) e bilateral (60%). A acufenometria revelou um pitch médio de 8,6 kHz e *loudness* médio de 14,1 dBNS. O incômodo causado pelo zumbido foi classificado como leve, com média de 25 pontos no THI.	O tipo de zumbido mais frequente é o de *pitch* agudo, contínuo e bilateral, 90% dos indivíduos apresentaram pelo menos um sinal ou sintoma de DTM e não há correlação do zumbido com a acufenometria, THI e checklist para DTM.
					Em relação à DTM, 90% dos participantes apresentaram ao menos um sinal ou sintoma, sendo o movimento de abertura de boca assimétrico o mais frequente (70%).	
		A idade média da amostra variou de 20 a 55 anos, com média de 32,1 anos.			Houve correlação estatisticamente significativa entre o *pitch* do zumbido e a *loudness* (quanto mais agudo o *pitch*, menor a loudness – P= 0,002); entre o *pitch* e o escore no THI ( P= 0,002 quanto mais agudo o *pitch,* maior o escore no THI). Não foram observadas correlações significativas entre os achados do checklist de DTM e os parâmetros psicoacústicos do zumbido ou o THI, sugerindo que, apesar da alta prevalência de sinais e sintomas de DTM na amostra, não foi identificada uma relação direta entre as características do zumbido e a DTM.	

Legenda: dB NA = *decibels* em nível de audição; dB NS = *decibels* em nível de sensação; DTM = Disfunção Temporo Mandibular; OD = Orelha direita; OE = Orelha esquerda; THI = *Tinnitus Handicap Inventory*

Os estudos selecionados para síntese confirmam que o zumbido é uma queixa comum em pacientes com DTM, mesmo na ausência de perda auditiva. Quanto aos estudos selecionados na presente revisão para a síntese, um deles^(10)^ apresenta que a maioria dos pacientes com DTM relatou zumbido unilateral, geralmente associado à sensação de plenitude auricular, mas sem otalgia. Apesar de apresentarem limiares auditivos dentro da normalidade, foram observadas alterações em exames complementares, como as emissões otoacústicas. Esses achados reforçam a necessidade de acompanhamento audiológico mesmo em pacientes sem perda auditiva.

Outra pesquisa^(13)^ identificou diferenças importantes nas características do zumbido em pacientes com e sem DTM. Indivíduos com DTM tendem a perceber o zumbido como um som único (em oposição a múltiplos sons), de menor intensidade e com *pitch* elevado, geralmente em torno de 6 kilohertz (kHz). Além disso, esses pacientes relataram maior frequência de modulação somatossensorial do zumbido, ou seja, conseguiram alterar a percepção do som por meio de movimentos mandibulares ou cervicais, um indicativo da contribuição do sistema somatossensorial na gênese do sintoma. Já outros autores^(12)^ identificaram que a maioria dos participantes relatou zumbido contínuo, localizado principalmente no ouvido direito, com *pitch* médio de aproximadamente 3.750 Hz e intensidade média entre 17 e 21 *decibels* em nível de sensação (dB NS). Os escores obtidos no *Tinnitus Handicap Inventory* (THI) indicaram um impacto moderado na qualidade de vida, sendo o desconforto mais acentuado em indivíduos com zumbido de maior duração e frequência. Também foi observada uma correlação positiva entre o tempo de duração do zumbido e os escores do THI.

Pesquisadoras^(11)^ que estudaram indivíduos com zumbido e audição normal observaram que 90% deles apresentavam pelo menos um sinal ou sintoma de DTM. O tipo mais frequente de zumbido foi do tipo agudo, contínuo e bilateral, com *pitch* médio elevado (8,6 kHz) e intensidade média de 14,1 dB NS. Apesar do incômodo relatado ter sido, em média, leve, verificou-se que quanto mais agudo o *pitch*, maior foi o escore no THI, sugerindo uma percepção mais incômoda. Além disso, o movimento de abertura bucal assimétrico foi o sinal de DTM mais frequentemente observado nesse grupo.

Outro estudo^(14)^ evidenciou que os pacientes com zumbido associado à DTM apresentaram características significativamente diferentes em relação ao grupo sem DTM. Um dos achados mais relevantes foi que metade dos pacientes com DTM relatou conseguir modular o zumbido por meio de movimentos da mandíbula ou do pescoço, enquanto apenas 21% dos pacientes sem DTM relataram o mesmo fenômeno (p = 0.001), o que sugere influência somatossensorial sobre a percepção do zumbido. Quando os pacientes com DTM foram subdivididos entre aqueles cuja queixa principal era o zumbido e aqueles que buscavam atendimento primariamente pela DTM, observou-se que o grupo queixoso de zumbido relatou maior impacto na qualidade de vida, evidenciado por escores mais altos no THI. No entanto, não houve outras diferenças significativas entre esses subgrupos, indicando que os parâmetros clínicos analisados não determinam, por si só, qual sintoma é mais incômodo para o paciente.

### Avaliação do risco de viés

Com o objetivo de mensurar a qualidade metodológica dos estudos selecionados foi empregada a ferramenta *Joanna Briggs Critical Appraisal* ^(15)^ com o checklist apropriado para cada desenho de estudo: os estudos transversais foram analisados a partir dos nove domínios da ferramenta *JBI Critical Appraisal Checklist for studies reporting prevalence data* (Tabela 2) e o estudo caso-controle foi avaliado conforme os dez domínios específicos da ferramenta *JBI Critical Appraisal Checklist for case control studies* (Tabela 3). O *software* Robvis^(16)^ foi usado para gerar a figura de risco de viés (Figura 2).

**Tabela 2:** Avaliação do risco de viés dos estudos transversais incluídos, segundo *JBI Critical Appraisal Tools*

Domínio JBI	Morais e Gil^(11)^	Lacerda et al.^(12)^	Cassol et al.^(10)^	Kanji e Khoza-Shangase^(13)^
D1 – Critérios de inclusão claramente definidos	Sim	Sim	Sim	Sim
D2 – Adequação do processo de recrutamento	Sim	Incerto	Sim	Sim
D3 – Tamanho amostral adequado	Não	Não	Sim	Sim
D4 – Descrição detalhada dos participantes e cenário	Não	Não	Sim	Sim
D5 – Cobertura adequada da amostra	Sim	Sim	Sim	Sim
D6 – Validade dos métodos de identificação da condição	Sim	Sim	Sim	Sim
D7 – Mensuração válida e confiável dos desfechos	Sim	Sim	Sim	Sim
D8 – Análise estatística apropriada	Sim	Sim	Sim	Sim
**Julgamento Global**	Moderado	Moderado-Alto	Baixo	Baixo

Legenda: D = domínio

**Tabela 3:** Avaliação do risco de viés do estudo caso-controle incluído, segundo *JBI Critical Appraisal Tools*

Domínio JBI	Vielsmeier et al.^(14)^
D1 – Critérios de caso claramente definidos	Sim
D2 – Representatividade dos casos	Sim
D3 – Seleção adequada dos controles	Sim
D4 – Comparabilidade entre casos e controles	Sim
D5 – Identificação válida da condição (casos)	Sim
D6 – Identificação válida da condição (controles)	Sim
D7 – Medição padronizada dos desfechos	Sim
D8 – Tratamento de fatores de confusão	Incerto
D9 – Análise estatística apropriada	Sim
Julgamento Global	Baixo

Legenda: D = domínio

**Figura 2:** Avaliação do risco de viés dos estudos incluídos na síntese, avaliados pelas ferramentas *JBI Critical Appraisal Tools*. A cor verde indica um baixo risco de viés, a amarela indica um risco incerto de viés e a vermelha indica um alto risco de viés. (A) Estudos transversais; (B) Estudos de caso-controle

Quanto aos estudos transversais, no escopo geral da análise, os estudos evidenciaram baixo risco de viés nos domínios relativos à definição e representatividade da população, aos procedimentos diagnósticos empregados e à mensuração das variáveis audiológicas e somatossensoriais. A maior parte dos domínios avaliados foram classificados como “baixo risco”, indicando adequada consistência metodológica nesses aspectos.

Entretanto, alguns domínios apresentaram inconsistências relevantes, particularmente entre os estudos transversais. O domínio referente ao tamanho amostral (D3) recebeu julgamento de alto risco em Morais e Gil^(11)^ e Lacerda et al.^(12)^, refletindo o pequeno número de participantes e a ausência de justificativa baseada em cálculo amostral. Já o domínio referente à descrição detalhada do cenário e dos participantes (D4) também apresentou risco elevado nesses mesmos estudos, dada a caracterização limitada da amostra e do contexto clínico. No estudo de Lacerda et al.^(12)^ observou-se ainda um julgamento “incerto” para D2, decorrente da natureza retrospectiva da coleta e da ausência de informações claras sobre o processo de recrutamento. O estudo de Kanji e Khoza-Shangase^(13)^ apresentou classificações favoráveis em praticamente todos os domínios, com exceção do tamanho amostral (D3), que recebeu avaliação “incerta” devido à ausência de justificativa formal, mas sem configuração clara de alto risco. Em relação ao estudo de caso-controle, observou-se consistente desempenho metodológico, com baixo risco em todos os domínios, exceto D2 e D9, ambos classificados como “incerto”, relacionados à descrição limitada do pareamento e do tempo de exposição. Os casos e controles foram comparados em diversas características, contudo, não foram descritas estratégias claras para evitar viés de seleção, ou para garantir representatividade entre os grupos. Embora os critérios para identificação de casos e controles tenham sido padronizados, utilizando diagnósticos baseados em análise funcional do sistema mastigatório, não houve uma descrição minuciosa sobre estratégias de pareamento entre os grupos, o que pode oferecer vieses na análise comparativa. Os resultados da avaliação crítica indicam que, apesar da heterogeneidade observada entre os estudos incluídos, não foram identificados padrões de viés a ponto de comprometer as conclusões gerais desta revisão. Ainda assim, os domínios com maior fragilidade metodológica foram cuidadosamente considerados durante a interpretação dos achados.

Foi realizada uma análise de sensibilidade de caráter qualitativo, com o propósito de examinar se as tendências observadas na síntese permaneceriam estáveis após a retirada dos estudos com maior concentração de julgamentos de risco de viés. A exclusão dos estudos de Morais e Gil^(11)^ e Lacerda et al.^(12)^ não alterou substancialmente os padrões gerais descritos na revisão, sugerindo que as conclusões preliminares não dependeram exclusivamente desses estudos. Ainda assim, tais resultados devem ser interpretados com cautela, dado o número reduzido de estudos e a heterogeneidade metodológica entre eles.

### Síntese dos resultados

Em síntese, os estudos analisados mostram que os pacientes com DTM geralmente apresentam zumbido de alta frequência, contínuo^(11)^, predominantemente unilateral (embora também possa ser bilateral), com intensidade superior a 10dBNS. O zumbido pode ser modulável por estímulos somatossensoriais e apresenta impacto leve a moderado na qualidade de vida^(13)^. A presença de zumbido em indivíduos com audição normal e sinais de DTM reforça a importância de uma avaliação multidisciplinar e de uma abordagem integrada entre fonoaudiologia, odontologia e outros profissionais da área da saúde. A síntese quantitativa dos cinco estudos incluídos revelou tendência de *pitch* predominantemente agudo, com média aproximada de 6.790 Hz (variando de 3.762 a 8.600 Hz) entre os estudos que realizaram acufenometria. A *loudness* média situou-se entre 14,1 e 33,5 dB NS, com valor central aproximado de 19,6 dB NS. Quanto à lateralidade, observou-se predomínio de zumbido unilateral nos pacientes com DTM, especialmente no estudo de Cassol et al.^(10)^, no qual 98% dos casos apresentavam correspondência entre o lado da DTM e o lado do zumbido. Nos estudos que relataram distribuição bilateral, a média aproximada foi de 60% bilateral^(11)^, enquanto Lacerda et al.^(12)^ indicou predomínio à direita (42%). Em relação à modulação somatossensorial, o estudo caso-controle de Vielsmeier et al.^(14)^ demonstrou que 66% dos indivíduos com DTM apresentavam alteração do zumbido mediante movimentos mandibulares ou cervicais, reforçando a participação dos mecanismos somatossensoriais na fisiopatologia. Os estudos analisados apresentaram como pontos fortes o uso de instrumentos validados, como o THI, a acufenometria e abordagens metodológicas detalhadas^(10-13)^. No entanto, limitações importantes foram observadas, principalmente no que diz respeito ao tamanho da amostra e às estratégias de seleção dos participantes. Portanto, embora os estudos tragam contribuições relevantes para o entendimento da relação entre zumbido e DTM, é necessário que mais estudos primários sejam realizados com tamanhos amostrais maiores e com a utilização de métodos claros de amostragem. Tais melhorias são fundamentais para aumentar a confiabilidade e a aplicabilidade das evidências geradas.

Ademais, verifica-se que dos cinco estudos selecionados para a síntese, apenas um estudo^(10)^, por exemplo, realizou audiometria tonal de altas frequências (AAF). No *hotmap,* apresentado na Figura 3 abaixo, cada estudo foi categorizado segundo três possíveis condições metodológicas referentes aos procedimentos audiológicos: “sim” (procedimento explicitamente realizado), “não” (procedimento explicitamente não realizado) e “não mencionado” (ausência de dessa informação no estudo). Essas categorias foram convertidas em tonalidades graduais de azul, nas quais cores mais escuras indicam maior completude e clareza metodológica (itens reportados de forma explícita) e tons mais claros representam lacunas de informação. Tal codificação cromática permite visualizar a heterogeneidade entre os estudos, tanto em relação à presença ou ausência de procedimentos específicos quanto ao grau de transparência na sua descrição.

**Figura 3:** *Hotmap:* Avaliações audiológicas e instrumentos utilizados

Legenda: *Hotmap* dos procedimentos audiológicos relatados nos estudos incluídos. As tonalidades de azul representam a presença, ausência ou ausência de menção de cada procedimento em cada estudo: Azul escuro (sim): o procedimento foi claramente descrito e realizado pelo estudo: Azul médio (*Não mencionado)*: o estudo não cita o procedimento, impossibilitando confirmar sua realização; Azul claro (*Não*): o estudo declara explicitamente que não realizou o procedimento. Os procedimentos mapeados foram: Emissões Otoacústicas (EOA), Acufenometria, Audiometria tonal limiar convencional (AT-AC), Audiometria de altas frequências (AAF) e Tinnitus Handicap Inventory (THI). A figura permite visualizar quais avaliações foram conduzidas em cada estudo e identificar padrões de utilização dos instrumentos audiológicos

## DISCUSSÃO

A presente revisão sistemática teve como objetivo identificar e descrever as características do zumbido associadas à disfunção temporomandibular (DTM), levando em consideração a relação já estabelecida entre essas duas condições^(17,18)^.

Os achados de Ren e Isberg^(19)^ mostraram que em todos os pacientes avaliados, o zumbido unilateral ocorreu no mesmo lado do deslocamento discal da ATM, o que corrobora a postulação de Costen^(20)^, que propôs que alterações mecânicas da ATM poderiam comprometer o nervo auriculotemporal, ramo sensitivo do trigêmeo que inerva tanto a cápsula articular quanto estruturas do meato acústico, além de modificar a pressão intratimpânica, gerando sintomas otológicos ipsilaterais. Estudos de revisão como as de Ralli et al.^(21)^ e Michiels et al.^(22)^ destacam que as vias somatossensoriais trigeminais e cervicais convergem precocemente no sistema auditivo, modulando a excitabilidade do núcleo coclear dorsal (NCD) e influenciando a integração entre aferências auditivas e somáticas. Esses modelos neurofisiológicos, somados à observação de que lesões somáticas podem desencadear zumbido ipsilateral, oferecem suporte para a hipótese de que mecanismos somatossensoriais possam contribuir para a percepção do zumbido no mesmo lado da disfunção.

Ainda assim, a força dessa associação permanece limitada pelo tamanho reduzido das amostras e pela heterogeneidade dos critérios diagnósticos empregados. Assim, há a necessidade de estudos com maior poder amostral e com avaliação padronizada da lateralidade associada à DTM.

Após a análise dos dados, evidenciou-se que o zumbido associado à DTM é caracterizado por ocorrer geralmente em *pitch* mais elevado, em torno de 6kHz. Apresenta também, *loudness* moderada, comumente menos intenso do que nos casos de pacientes com zumbido cujos sintomas são originados por outros fatores que não a DTM^(23)^.

No que diz respeito aos achados da presente revisão de *pitch* em altas frequências, *loudness* moderada e zumbido somatossensorial, a literatura mostra a convergência precoce de aferências somatossensoriais trigeminais e cervicais sobre o núcleo coclear dorsal (DCN) que modulam sua excitabilidade e desencadeiam a plasticidade mal-adaptativa nos circuitos auditivos centrais, mesmo em indivíduos com audição periférica normal ou apenas levemente alterada^(21,22,24)^. Evidências experimentais indicam que, diante de irritação somática persistente ou de mínima privação auditiva, o DCN exibe aumento das taxas de disparo, maior sincronização neuronal e intensificação da influência excitatória proveniente das vias somatossensoriais^(25)^, que tende a afetar preferencialmente regiões tonotópicas de alta frequência. Além disso, como esses pacientes frequentemente mantêm audição periférica relativamente preservada, o zumbido resulta mais de modulação somatossensorial do que de um dano coclear. Nesses casos, a atividade desregulada é suficiente para gerar a percepção do sintoma, porém sem grande amplificação central, o que justificaria a *loudness* moderada^(24)^.

Embora as características apresentadas neste estudo de revisão possam sugerir um atributo específico do zumbido relacionado à DTM, é importante destacar que outras condições clínicas, como perda auditiva oculta e perda auditiva induzida por ruído (PAIR), também podem apresentar zumbido com *pitch* em torno de 6kHz^(26,27)^. Essa sobreposição de características entre diferentes patologias dificulta a identificação de um tipo único, ou distintivo de zumbido associado exclusivamente à DTM. Assim, estabelecer um perfil sonoro específico, ou uma característica pontual do zumbido que possa ser considerada um indicativo claro e exclusivo da presença de DTM torna-se inconclusivo^(10,13)^.

A diferenciação entre zumbido associado à DTM e zumbido induzido por ruído, no entanto, tem implicações clínicas importantes. O zumbido relacionado à DTM tende a ocorrer em indivíduos com audição periférica normal, ou apenas levemente alterada, e pode frequentemente ser acompanhado por modulação somática e/ou dor orofacial^(22)^. Por outro lado, grandes estudos populacionais sobre exposição ocupacional ao ruído mostram que o zumbido induzido por ruído é mais prevalente entre trabalhadores expostos cronicamente e está fortemente associado à perda auditiva neurossensorial, especialmente nas frequências entre 3 e 6 kHz^(28)^. Esse contraste reforça a relevância de considerar o perfil audiológico, o contexto ocupacional e a presença de sensibilidade somática ao classificar subtipos de zumbido na prática clínica.

Em pacientes mais jovens com zumbido associado à DTM, observa-se que a sensação pode ser aliviada por meio de movimentos da mandíbula, cabeça ou pescoço. Nesses casos, fatores de risco clássicos para o zumbido, como idade, sexo masculino e perda auditiva, mostram-se menos relevantes. Com base nisso, o tratamento da DTM pode representar uma abordagem terapêutica direcionada à causa do zumbido^(14,19)^. Esses achados evidenciam, ainda, a relevância de uma avaliação criteriosa e de uma abordagem multidisciplinar no manejo do zumbido, incluindo, por exemplo, a atuação de profissionais da odontologia^(14)^, considerando que essa condição pode impactar significativamente tanto o diagnóstico quanto a condução terapêutica.

Os resultados também indicaram que a presença de alterações auditivas em pacientes com DTM é significativa. Portanto, é fundamental que esses pacientes sejam informados sobre as possíveis causas dessa condição. Além disso, deve ser recomendado acompanhamento audiológico aos indivíduos com DTM, independente da presença de zumbido, a fim de monitorar a evolução e possibilitar intervenções adequadas^(19)^.

A constatação de que metade dos estudos selecionados para leitura na íntegra são produções brasileiras^(10-12)^ evidencia a relevância do assunto de pesquisa no contexto nacional. Portanto, mostra o avanço de pesquisas audiológicas do Brasil no cenário mundial. Essa observação sugere adequação metodológica e cultural nas pesquisas sobre zumbido, refletindo uma tendência, especialmente no contexto brasileiro, de se privilegiar a investigação de aspectos psicoacústicos.

Embora tais estudos apresentem contribuições importantes, especialmente na caracterização psicoacústica do zumbido associado à DTM, algumas limitações devem ser reconhecidas. Entre elas, destacam-se o tamanho reduzido das amostras^(13)^, e a adoção de protocolos avaliativos restritos. Em vários casos, as investigações utilizaram exclusivamente medidas subjetivas de impacto, como o *Tinnitus Handicap Inventory* (THI), sem incorporar avaliações audiológicas mais abrangentes, incluindo acufenometria, testes específicos de modulação somatossensorial e audiometria em altas frequências. Essas restrições metodológicas limitam a comparabilidade entre estudos e reduzem a capacidade de estabelecer conclusões mais robustas sobre o perfil psicoacústico do zumbido em pacientes com DTM.

Além disso, há heterogeneidade nos critérios diagnósticos de DTM, na padronização dos procedimentos de acufenometria e nos instrumentos utilizados para quantificação do incômodo, como o THI. O relato incompleto de procedimentos audiológicos complementares também dificulta a comparação entre estudos e limita inferências sobre a relação entre integridade coclear e características do zumbido.

Apesar de três estudos apresentarem risco de viés classificado como baixo nas ferramentas da JBI, dois apresentaram viés moderado, particularmente nos domínios de validade externa e controle de fatores de confusão. Assim, embora os resultados sejam consistentes entre si, a certeza global da evidência é limitada e reforça a necessidade de estudos longitudinais com delineamentos mais robustos, padronização nos critérios diagnósticos e maior controle de variáveis audiológicas e somatossensoriais.

Os dados reforçam a importância de protocolos audiológicos e psicoacústicos padronizados para pacientes com DTM, incluindo testes de *pitch matching* e *loudness matching*. A compreensão desses parâmetros pode auxiliar na diferenciação de subtipos de zumbido somatossensorial e no direcionamento de terapias integradas, envolvendo motricidade orofacial, reabilitação auditiva, fisioterapêutica e odontológica.

## CONCLUSÃO

Os resultados obtidos na revisão sistemática evidenciaram que o zumbido apresentado pelos pacientes com diagnóstico de DTM é contínuo, geralmente unilateral e associado ao lado da disfunção, de *pitch* elevado, com média de frequência variando entre 3.750Hz e 8.600Hz e média de *loudness* entre 14dBNS e 25dBNS. Pode estar associado ou não à presença de perda auditiva, sendo que mesmo nos casos de limiares auditivos normais, sugere-se controle audiológico. Verificou-se impacto moderado na qualidade de vida, com associação entre o tempo de presença do sintoma, o *pitch* e a pontuação obtida no instrumento específico de avaliação da qualidade de vida. Um fato muito importante é que os pacientes com DTM apresentaram modulação do zumbido com a realização de movimentos mandibulares e cervicais, o que os diferenciou dos casos em que o zumbido não estava associado à disfunção pesquisada.

## Apêndice A Estratégias de busca

**Table:**

**BASE DE DADOS**	**ESTRATÉGIA DE BUSCA**
PUBMED	("*temporomandibular joint disorders*" [MeSH Terms] OR "*Temporomandibular Disorders*" OR "*Temporomandibular Joint Disorder*" OR "*Temporomandibular Joint Diseases*" OR "*Temporomandibular Disorders*") AND ("*tinnitus*" [MeSH Terms] OR tinnitus) AND (*pitch* [All Fields] OR *loudness* [All Fields] OR *characteristics* [All Fields])
EMBASE	('*temporomandibular joint disorders*' OR '*Temporomandibular Disorders*' OR '*Temporomandibular Joint Disorder*' OR '*Temporomandibular Joint Diseases*' OR '*Temporomandibular Disorders*') AND ('*tinnitus*' or*tinnitus*) AND (*pitch* OR *loudness* OR *characteristics*)
LILACS	(MH:"*Temporomandibular Joint Dysfunction Syndrome*" OR "*Temporomandibular Joint Dysfunction Syndrome*" OR "Síndrome da Disfunção da Articulação Temporomandibular" OR "*Síndrome de la Disfunción de Articulación Temporomandibular*" OR "*Síndrome Miofascial de Disfunção Dolorosa Temporomandibular*" OR "Síndrome da ATM" OR "Síndrome da Articulação Temporomandibular" OR MH: C05.500.607.221.897.897 OR MH: C05.550.905.905 OR MH: C05.651.243.897.897 OR MH: C05.651.550.905 OR MH: C07.320.610.291.897.897 OR MH: C07.678.949) AND (MH:"*tinnitus*" OR "*tinnitus*" OR "zumbido" OR "*acúfeno*" OR "*tinido*" OR "zumbido pulsátil" OR "*zumbidos*" OR "*zunido*" OR MH:C09.218.458.670 OR MH:C10.597.751.418.670 OR MH: C23.888.592.763.393.670)
Web Of Science	("*temporomandibular joint disorders*" OR "*Temporomandibular Disorders*" OR "*Temporomandibular Joint Disorder*" OR "*Temporomandibular Joint Diseases*" OR "*Temporomandibular Disorders*") AND ("*tinnitus*") AND (*pitch* OR *loudness* OR *characteristics*)
Scopus	("*temporomandibular joint disorders*" OR "*Temporomandibular Disorders*" OR "*Temporomandibular Joint Disorder*" OR "*Temporomandibular Joint Diseases*" OR "*Temporomandibular Disorders*") AND ("*tinnitus*" OR *tinnitus*) AND (*pitch* OR *loudness* OR *characteristics*)
Google Scholar	"*temporomandibular joint disorders*" OR "*Temporomandibular Disorders*" OR "*Temporomandibular Joint Disorder*" OR "*Temporomandibular Joint Diseases*" OR "*Temporomandibular Disorders*" AND "*tinnitus*" AND *pitch* OR *loudness* OR *characteristics*filetype:pdf

## Apêndice B Estudos excluídos com os respectivos motivos para exclusão (n=13)

**Table:**

**Autor, Ano**	**Razão de Exclusão**
Pavaci et al., 2019	1
Mazzetto et al., 2013	2
Bousema et al., 2018	3
Michiels et al., 2018	2
Pozuelo-Pinilla et al., 2010	3
Michiels, 2020	3
Mahmoudian et al., 2022	4
Giordani, 1993	2
Clauser et al., 2011	2
Upton e Wijeyesakere, 2004	2
Sanchez et al., 2007	1
Vielsmeier et al., 2012	1
van Oijen, 2022	1

1 Estudos que não investigaram o desfecho de interesse

2 O estudo não apresenta dados psicoacústicos sobre o zumbido

3 Estudo de revisão

4 Estudo do tipo série de casos
